# Supplementary material for: Gut mucin fucosylation dictates the entry of botulinum toxin complexes
Source: Nat Commun. 2025 Nov 25;16:10442. doi: 10.1038/s41467-025-65384-w (PMC12647801; doi:10.1038/s41467-025-65384-w)
Supplement: Supplementary file 1 — Supplementary Information [file 41467_2025_65384_MOESM1_ESM.pdf]

Supplementary Material for

Gut mucin fucosylation dictates the entry of botulinum toxin complexes

Sho Amatsu[#], Takuhiro Matsumura[#], Chiyono Morimoto, Sunanda Keisham, Yoshiyuki Goto, Tomoko Kohda, Jun Hirabayashi, Kengo Kitadokoro, Takane Katayama, Hiroshi Kiyono, Hiroaki Tateno, Masahiko Zuka, and Yukako Fujinaga[*]

# These authors contributed equally to this work

* For correspondence: Yukako Fujinaga

Supplementary Figs. 1–13

Supplementary Table 1

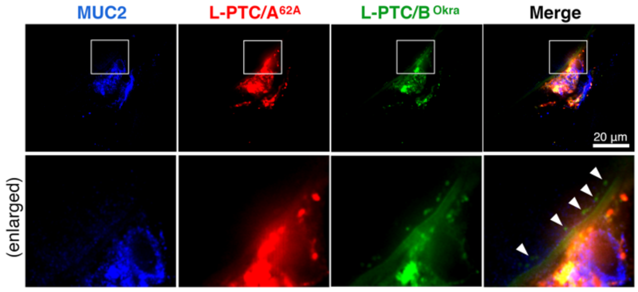

Supplementary Figure 1. *In situ* ligated mouse intestinal loop assay with L-PTC/A^{62A} and L-PTC/B^{Okra}.

Representative images of whole-mounted small intestine with large progenitor toxin complexes of botulinum toxin (L-PTCs). A mixture of Alexa Fluor (AF) 568–labeled L-PTC/A^{62A} (red) and AF 488–labeled L-PTC/B^{Okra} (green) was injected into ligated mouse intestinal loops. Mucin was visualized with an anti-MUC2 antibody (blue). The upper panel is also shown in Fig. 1c. The lower panel is an enlarged view of the square in the upper panel. L-PTC/B^{Okra} was absorbed from enterocytes in the villous epithelium (white arrowhead), whereas L-PTC/A^{62A} was trapped in the mucus. Scale bar, 20 µm.

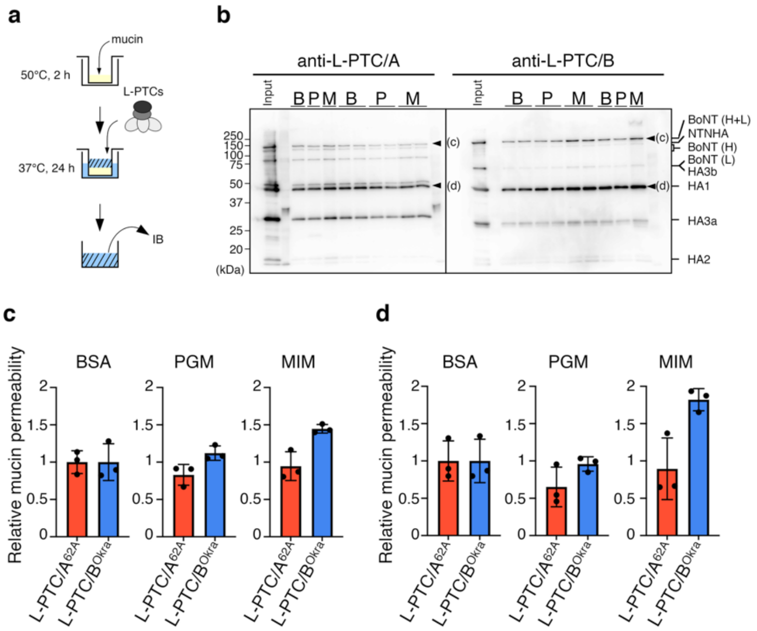

Supplementary Figure 2. Mucin penetration assay.

a, Bovine serum albumin (BSA), porcine gastric mucin (PGM), or mouse intestinal mucin (MIM) was coated on Transwell 24-well filters (0.4-µm pore) at 50°C for 2 h. The filters were incubated with 100 nM L-PTCs (upper chambers) at 37°C for 24 h. **b-d**, Upper-to-lower penetration of L-PTCs was assessed by immunoblotting using anti–L-PTC/A and anti–L-PTC/B antiserum and calculated by densitometric analysis. To compare the mucin permeability between A-62A and B-Okra, the band intensities of intact BoNT (H+L, heavy chain and light chain) (**c**) and HA1 (**d**) from PGM- and MIM-coated wells were normalized to those from BSA-coated wells. The permeability through PGM calculated using intact BoNT intensities (**c**) is the same figure shown in Fig. 1c. Values represent the mean ± SD of triplicate wells. B, BSA. P, PGM. M, MIM.

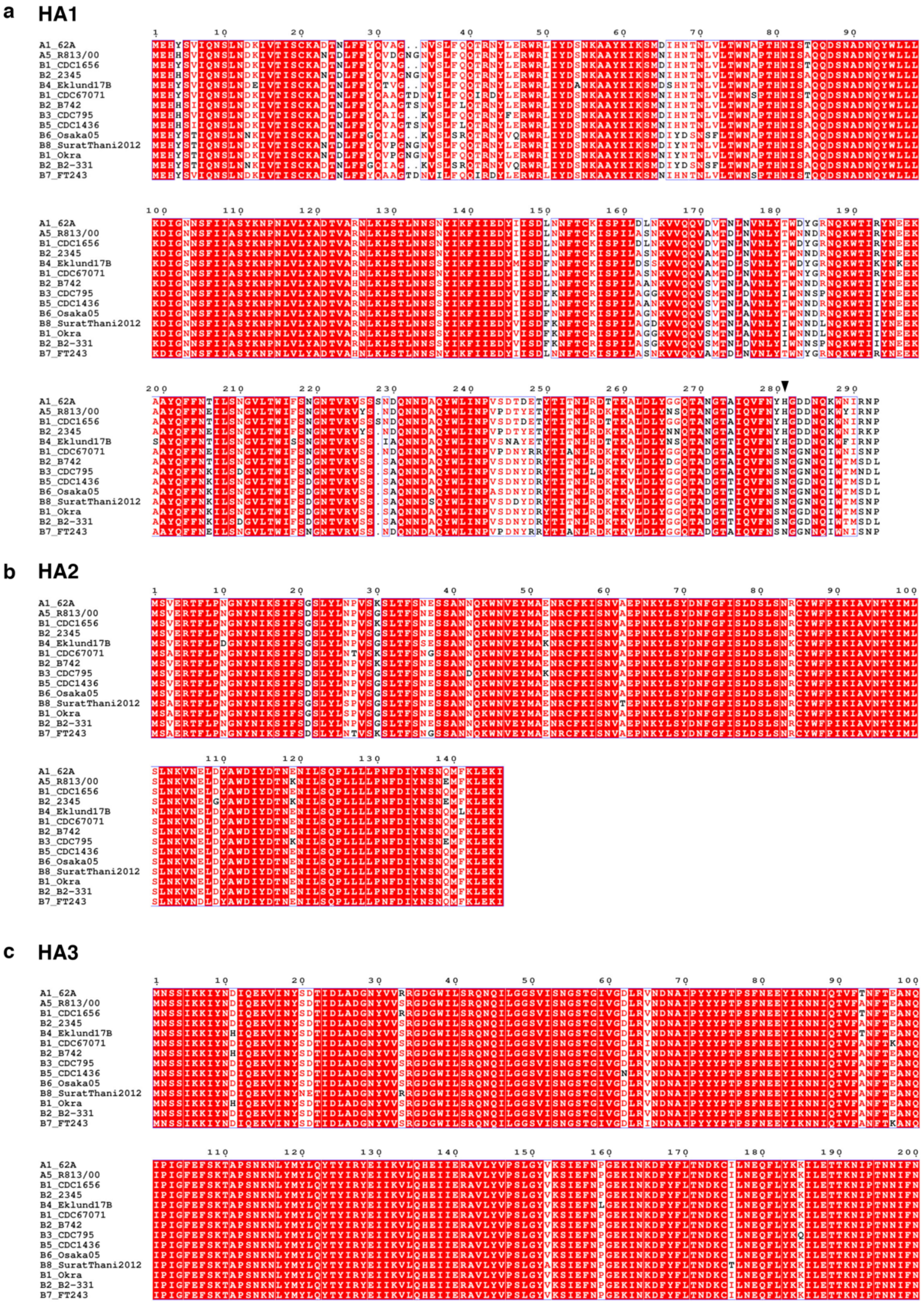
a
HA1
b
HA2
c
HA3
A1_62A
A5_R813/00
B1_CDC1656
B2_2345
B4_Eklund17B
B1_CDC67071
B2_B742
B3_CDC795
B5_CDC1436
B6_Osaka05
B8_SuratThani2012
B1_Okra
B2_B2-331
B7_FT243

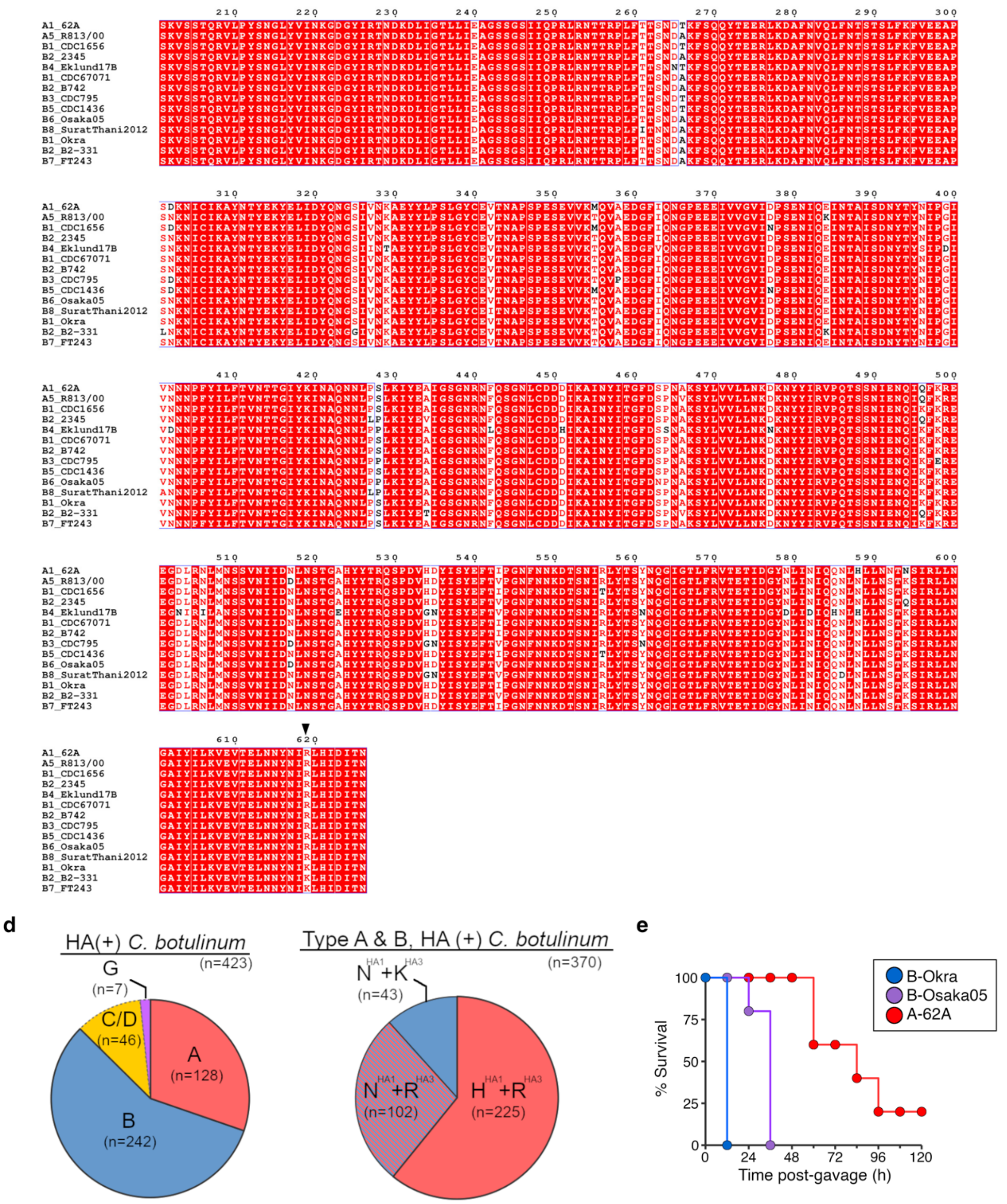

Supplementary Figure 3. Classification of HA by amino acid sequence.

a–c, Multiple sequence alignments of HA1 (**a**), HA2 (**b**), and HA3 (**c**). Black arrowheads, HA1$^{H281/N282}$ and HA3$^{R619/K619}$. **d**, Summary of HA sequence types from *C. botulinum* reported in the NCBI database (*n* = 423). Forty-three strains (11.6%) of the hyper-oral-

toxic sequence type of HA ($N282^{HA1}/K619^{HA3}$) are found in serotypes A and B (n = 370). **e**, Survival curves of BALB/c mice (n = 5 per group) challenged i.g. with 1 µg of L-PTC/B^{Okra} (HA sequence type: $N282^{HA1}/K619^{HA3}$), L-PTC/$B^{Osaka05}$ (HA sequence type: $N280^{HA1}/R619^{HA3}$), or L-PTC/A^{62A} (HA sequence type: $H281^{HA1}/R619^{HA3}$). The survival data of mice administered with L-PTC/B^{Okra} and L-PTC/A^{62A} are from the same experiment as Fig. 1a.

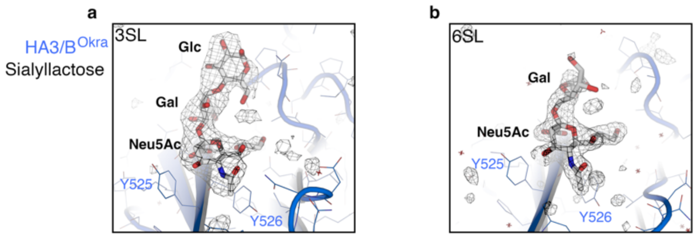

Supplementary Figure 4. Electron density for sialyllactose bound to HA3/B$^{\mathbf{Okra}}$.

The electron densities for α2,3-sialyllactose (3SL, **a**) and α2,6-sialyllactose (6SL, **b**) in the *mFo-DFc* omit maps (contoured at 3.0σ) are shown as gray mesh.

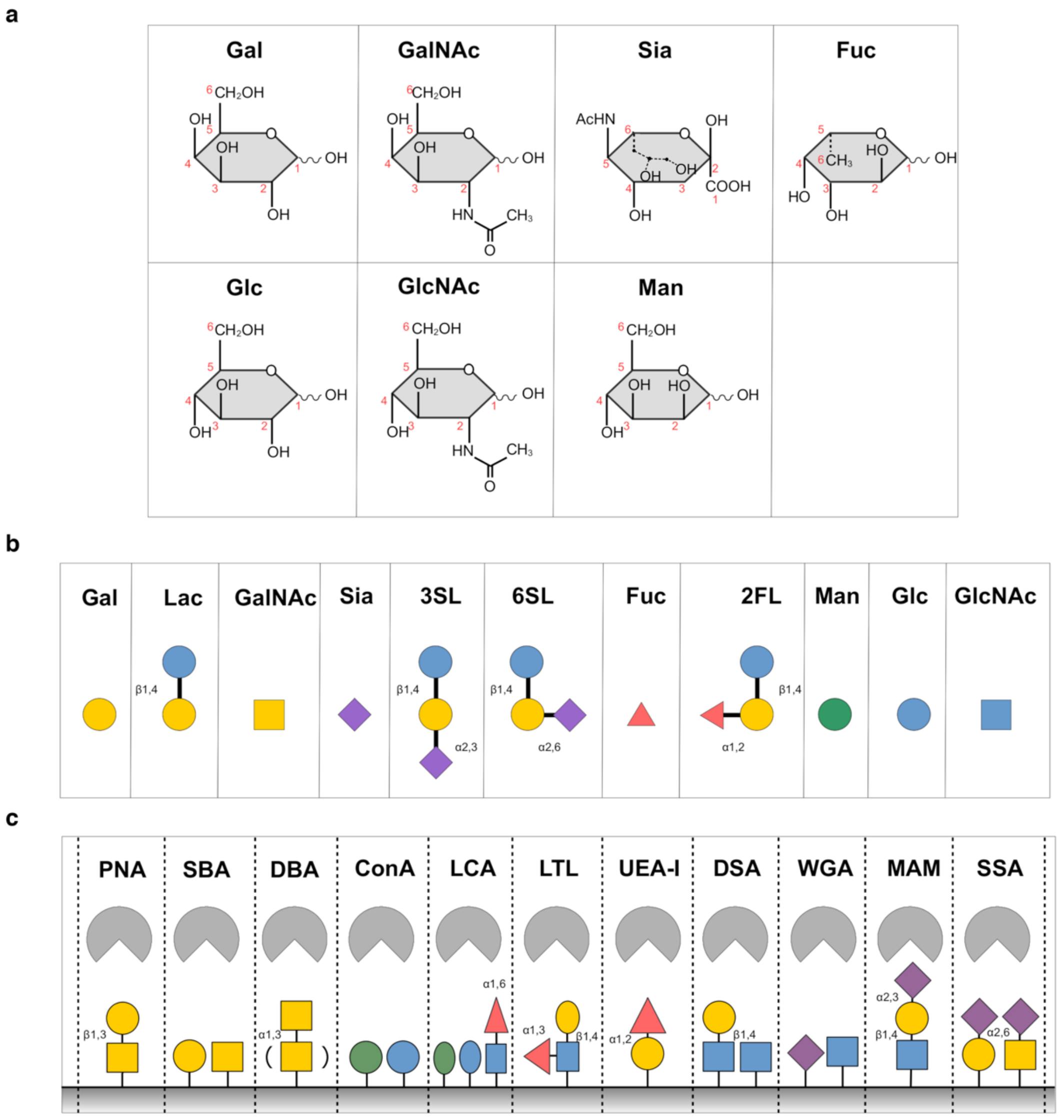

Supplementary Figure 5. Carbohydrates and lectins.

a, **b**, Structures of monosaccharides (D-galactose, *N*-acetyl-D-galactosamine, 5-*N*-acetyl-D-neuraminic acid, L-fucose, D-glucose, *N*-acetyl-D-glucosamine, D-Mannose) (**a**) and oligosaccharides (**b**). **c**, Binding specificity of lectins[1,2]. Gal, galactose; Lac, lactose; GalNAc, *N*-acetylgalactosamine; Sia, sialic acid; 3SL, α2,3-sialyllactose; 6SL, α2,6-sialyllactose; Fuc, fucose; 2FL, α1,2-fucosyllactose; Man, mannose; Glc, glucose; GlcNAc, *N*-acetylglucosamine; PNA, peanut agglutinin (Gal-β1,3-GalNAc–specific

lectin); SBA, soybean agglutinin (Gal– and GalNAc–specific lectin); DBA, *Dolichos biflorus* agglutinin (GalNAc-α–specific lectin); ConA, concanavalin A (Man– and Glc–specific lectin); LCA, *Lens culinaris* agglutinin (Man–, Glc–, and Fuc-α1,6–specific lectin); LTL, *Lotus tetragonolobus* agglutinin (Fuc-α1,3–specific lectin); UEA-I, *Ulex europaeus* agglutinin I (Fuc-α1,2-Gal–specific lectin); DSA, *Datura stramonium* agglutinin (Gal-β1,4-GlcNAc– and GlcNAc–specific lectin); WGA, wheat germ agglutinin (Sia– and GlcNAc–specific lectin); MAM, *Maackia amurensis* mitogen (3-*O*-sulfo-Gal-β1,4-GlcNAc– and Sia-α2,3-Gal-β1,4-GlcNAc–specific lectin); SSA, *Sambucus sieboldiana* agglutinin (Sia-α2,6-Gal– and Sia-α2,6-GalNAc–specific lectin).

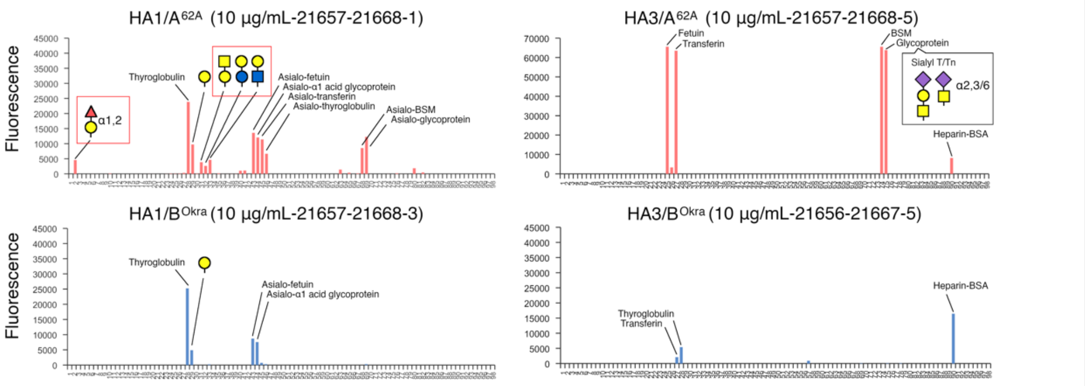

Supplementary Figure 6. Carbohydrate-binding specificities of HA1 and HA3.

The carbohydrate-binding specificities of HA1/A^{62A}, HA1/B^{Okra}, HA3/A^{62A}, and HA3/B^{Okra} were evaluated using a glycan microarray (Supplementary Table 2). Cy3–labeled 10 µg/mL HA subcomponent proteins were applied to the microarray plates. Oligosaccharide compositions are given as a red triangle (fucose), yellow circles (galactose), yellow squares (*N*-acetylgalactosamine), blue circle (glucose), blue square (*N*-acetylglucosamine), and purple diamonds (sialic acid).

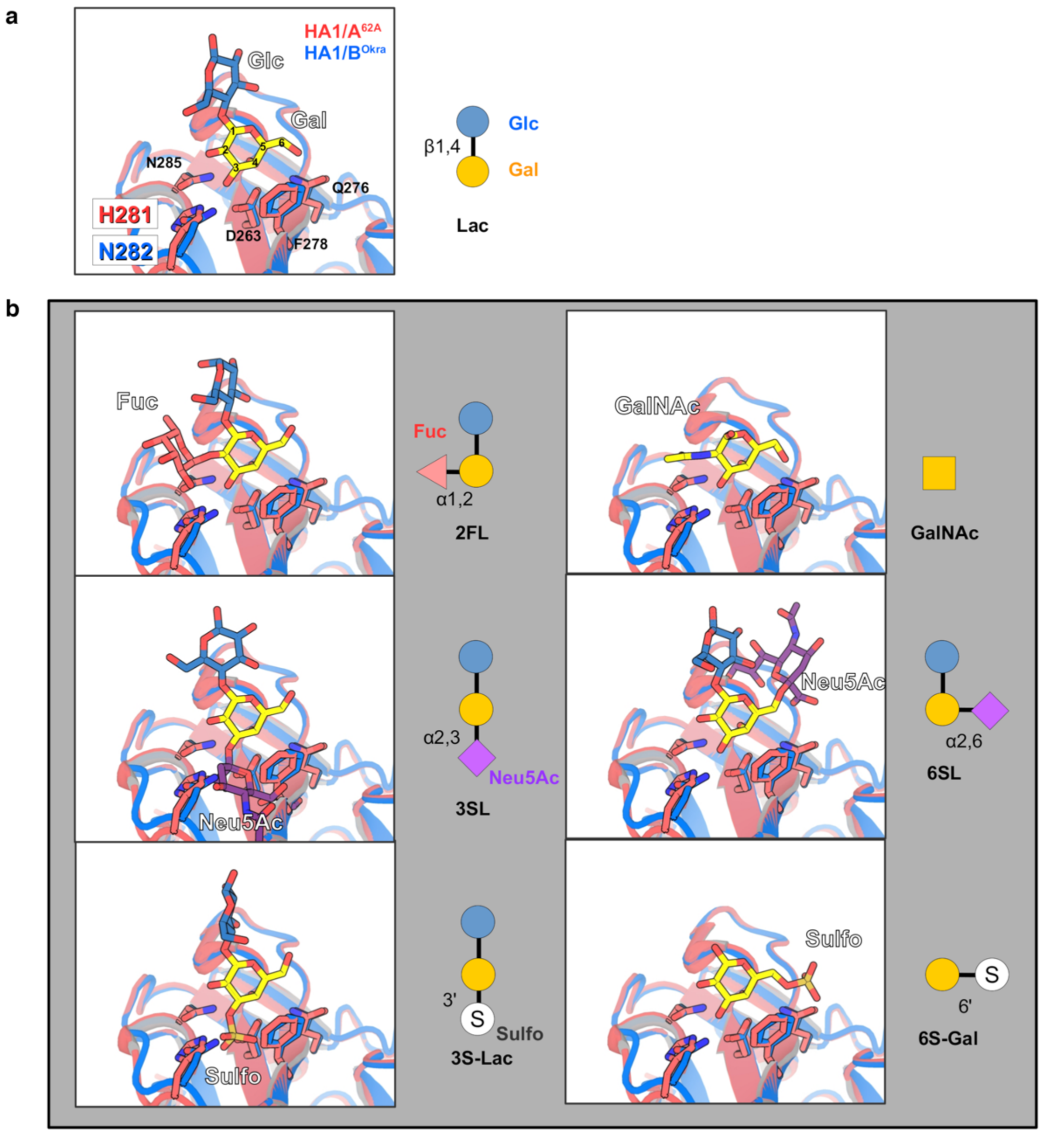

Supplementary Figure 7. Docking models of carbohydrates on HA1.

a, Crystal structures of HA1/A^{62A} and HA1/B^{Okra} with lactose (Lac, galactose-β(1,4)-glucose; ID: 4LO2[3], 4OUJ[4]). **b**, Carbohydrates were manually superimposed to the crystal structures; 2FL, α1,2-fucosyllactose; GalNAc, *N*-acetylgalactosamine; 3SL, α2,3-sialyllactose; 6SL, α2,6-sialyllactose; 3S-Lac; 3-*O*-sulfo-lactose, 6S-Lac; 6-*O*-sulfo-lactose.

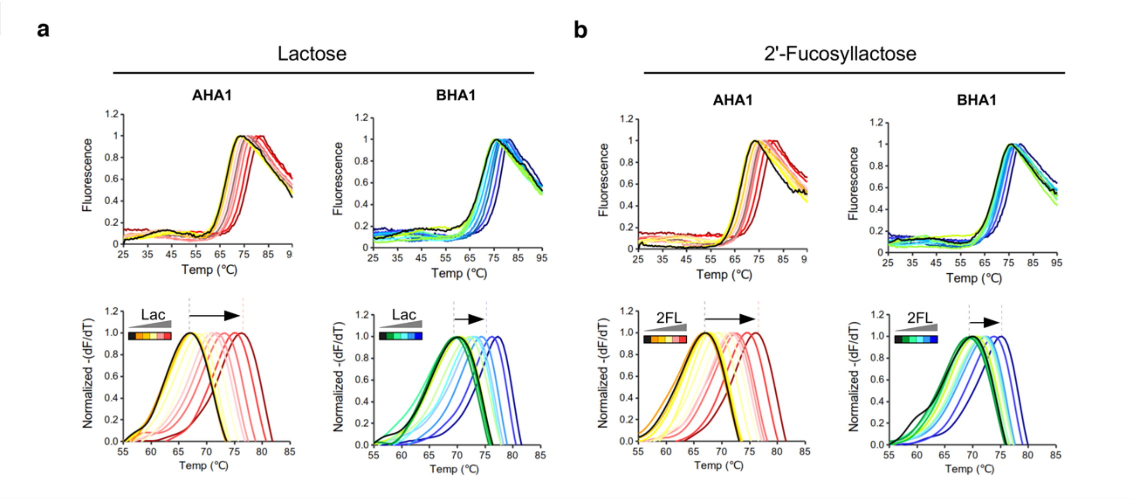

Supplementary Figure 8. Interactions between HA1 and α1,2-fucosyllactose.

a, **b**, HA1–lactose (Lac) (**a**) and HA1–α1,2-fucosyllactose (2FL) (**b**) interactions were analyzed using a thermal shift assay in quadruplicate. HA1/A^{62A} and HA1/B^{Okra} (2 µM in PBS (pH 7.4)) were mixed with 0–200 mM Lac or 2FL and melted in the presence of a dye (SYPRO Orange).

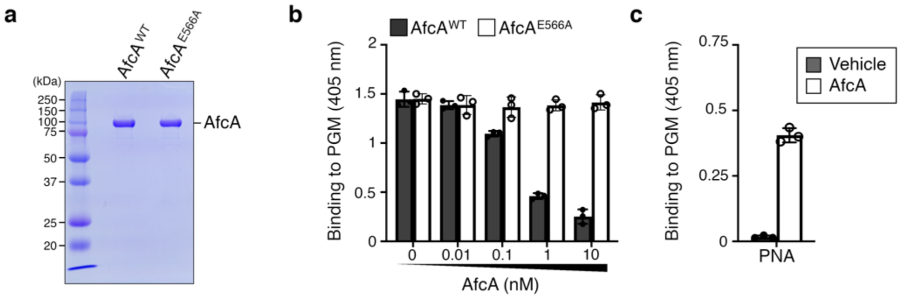

Supplementary Figure 9. Removal of terminal fucose from PGM with AfcA.

a, Preparation of recombinant wild-type α1,2-fucosidase AfcA (WT) and an inactive mutant (E566A). The SDS-PAGE gel was stained with Coomassie brilliant blue. **b**, **c**, Porcine gastric mucin (PGM) was treated with 0–10 nM WT (filled bar) or E566A (open bar) before lectin addition. Glycosylation of PGM was assessed with UEA-I (**b**; fucose-α(1,2)-galactose–specific lectin) and PNA (**c**; galactose-β(1,3)-*N*-acetylgalactosamine–specific lectin). Values represent the mean ± SD of triplicate wells.

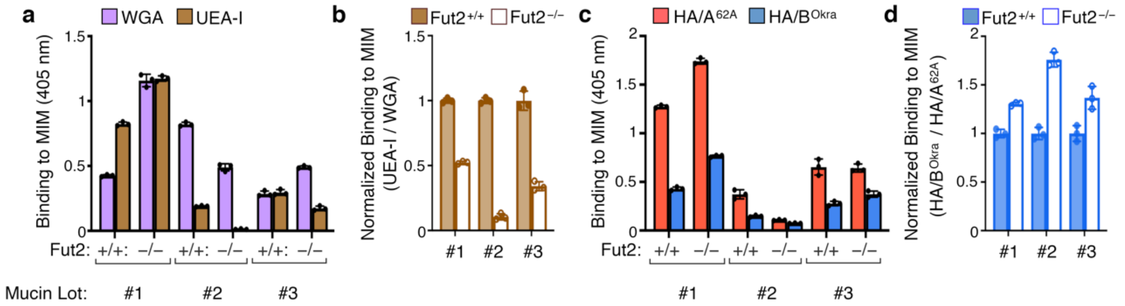

Supplementary Figure 10. Interaction between HA and mucin in *Fut2*-null mice.

a–**d**, Mucin ELISA with mouse intestinal mucin from WT (MIM^{WT}) and *Fut2*-null mice (MIM^{Fut2}) (n = 3; each experiment was performed in triplicate using MIM from three different mucin purification lots: #1, #2, #3). The glycosylation of MIM was assessed with WGA (purple, Sia– and GlcNAc–specific lectin) and UEA-I (brown, fucose-α(1,2)-galactose–specific lectin) (**a**), and the binding of UEA-I was normalized to that of WGA (**b**). The binding of HA/A^{62A} (red) and HA/B^{Okra} (blue) to MIMs was assessed (**c**) and the binding of HA/B^{Okra} was normalized to that of HA/A^{62A} (**d**). These data are summarized in Fig. 4b.

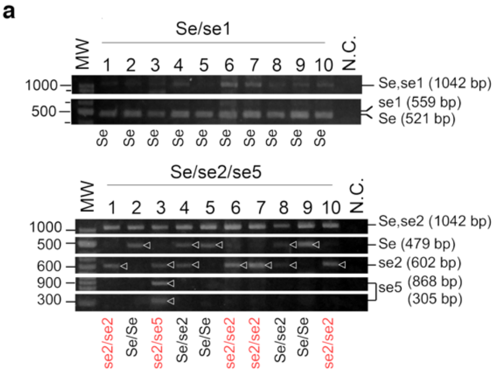

b

	Case No.	Sex	Age (yo)	Genotype
HIM01	021-37	Male	78	se2/se2
HIM02	021-46	Female	56	Se/Se
HIM03	021-53	Male	92	se2/se5
HIM04	021-54	Male	79	Se/se2
HIM05	021-65	Male	93	Se/Se
HIM06	021-74	Female	79	se2/se2
HIM07	021-92	Male	79	se2/se2
HIM08	021-96	Male	0 (4 mo)	Se/se2
HIM09	022-12	Male	0 (6 mo)	Se/Se
HIM10	022-32	Female	0 (4 mo)	se2/se2

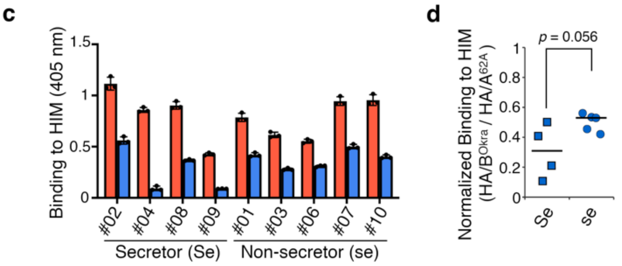

Supplementary Figure 11. The effect of *FUT2* secretor genotypes on the interaction between HA and HIM.

a, **b**, Human intestinal mucin (HIM) was isolated and purified from human mucus samples, and the genotypes (Se, secretor; non-secreters se1, G428A; se2, A385T; se5, fusion/del)[5] were identified by multiplex PCR (**a**). **b**, The table shows sample information and genotyping results. The non-secretors (HIM01, HIM03, HIM06, HIM07, HIM10) were indicated by red font. **c**, ELISA was used to analyze the binding of HA/A^{62A} (red) and HA/B^{Okra} (blue) to HIM. Values represent the mean ± SD of triplicate wells. The HA binding to HIM05 was not tested because of a failed attempt to purify the mucin. **d**, The binding of HA/B^{Okra} was normalized to that of HA/A^{62A}; two-tailed Student's *t*-test.

a

Type A

		Numbers of mice (alive/tested)				
		1	2	3	4	5 (day)
Non-labeled	100 pg	0/2				
Alexa-labeled	100 pg	2/2	2/2	2/2	2/2	2/2
	500 pg	2/2	2/2*	2/2*	2/2*	2/2
	750 pg	2/2*	2/2*	2/2*	1/2*	1/2*
	1000 pg	0/2				

*Mice show botulism symptoms

b

Type B

		Numbers of mice (alive/tested)				
		1	2	3	4	5 (day)
Non-labeled	100 pg	0/2				
Alexa-labeled	100 pg	2/2	2/2	2/2	2/2	2/2
	500 pg	2/2*	2/2*	2/2*	2/2*	2/2*
	750 pg	2/2*	2/2*	1/2*	1/2*	1/2*
	1000 pg	0/2				

*Mice show botulism symptoms

Supplementary Figure 12. The toxicity of the Alexa Fluor-labeled L-PTCs.

Female BALB/c mice (two mice per group) were injected intraperitoneally with the indicated concentrations of non-labeled or Alexa Fluor-labeled toxins of A-62A (**a**) and B-Okra (**b**). The presence of botulism symptoms in mice during the observation period is indicated by asterisks.

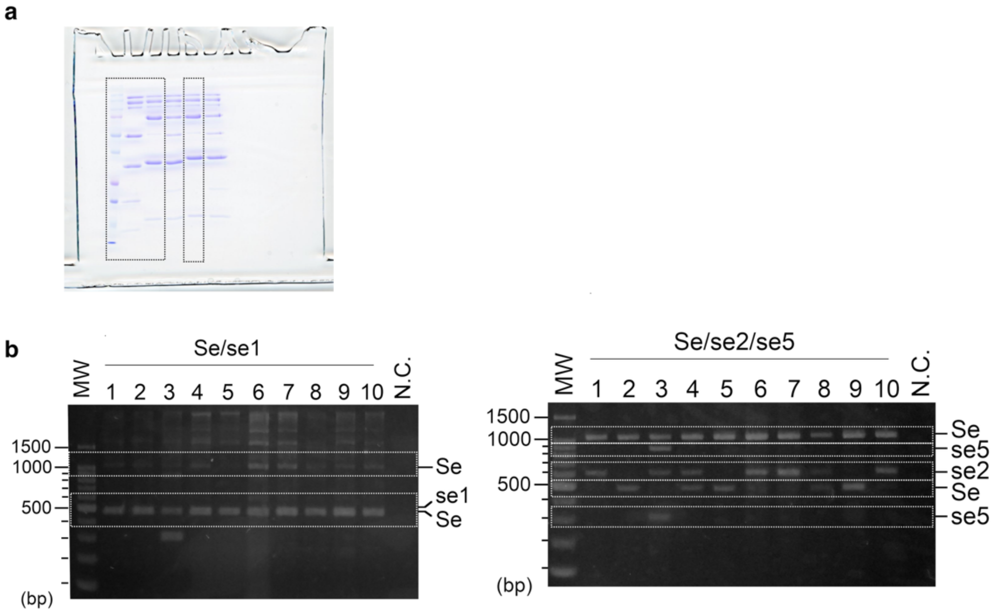

Supplementary Figure 13. Uncropped images.

Uncropped images of SDS-PAGE of rL-PTCs shown in Fig. 2d (**a**) and FUT2 genotyping shown in Supplementary Fig. 10a (**b**). Dot line boxes indicate cropped lines.

Supplementary Table 1. Data collection and refinement statistics

	HA3/B^{Okra}–3SL (PDB: 9UG5)	HA3/B^{Okra}–6SL (PDB: 9UG6)
**Diffraction data**		
X-ray source	SPring-8 / BL44XU	
Wavelength (Å)	0.800	0.800
Resolution range (Å)	41.44–3.00 (3.11–3.00)	42.08–2.23 (2.31–2.23)
Space group	$P2_12_12_1$	$P2_12_12_1$
a, b, c (Å)	82.0, 144.1, 158.0	84.3, 145.7, 159.7
α, β, γ (°)	90, 90, 90	90, 90, 90
Unique reflections	34,416 (3,312)	91,280 (8,958)
Completeness (%)[a]	90.0 (87.9)	94.8 (94.5)
Mean *I/sigma (I)*	7.1 (3.1)	13.8 (5.1)
R_{merge} (%)[b]	10.5 (50.1)	4.7 (21.4)
Multiplicity	3.6 (3.5)	3.8 (3.3)
**Refinement**		
R-factor[c] / R_{free}[d] (%)	22.4 / 30.3	22.9 / 28.7
No. of molecules per asymmetric unit	3	3
No. of protein atoms		
Protein	13,576	13,958
Ligand/Ion	86	52
Solvent	16	190
B-factors (Å^2)		
Protein	64.2	25.1
Ligand/Ion	66.2	29.2
Solvent	31.3	33.0
Ramachandran distribution		
Favored (%)	94.3	95.6
Allowed (%)	5.1	4.3
Outliers (%)	0.7	0.1
r.m.s.d.[e]		
Bond length (Å)	0.016	0.009
Angles (°)	1.6	1.8

a. Completeness for all reflections and for the highest resolution shell is shown in parentheses.
b. $R_{merge} = \Sigma|Ii - \langle Ii\rangle| / \Sigma\langle Ii\rangle$, where Ii is the observed intensity, and ⟨Ii⟩ is the average intensity over symmetry equivalent measurements.
c. *R*-factor = $\Sigma||F_{obs}| - |F_{calc}|| / \Sigma|F_{obs}|$.
d. R_{free} is calculated as *R*-factor but on 5% of all reflections that were never used in crystallographic refinement.
e. r.m.s.d., root mean square deviation; PDB, Protein Data Bank.

References

1. Bojar, D. *et al.* A Useful Guide to Lectin binding: Machine-learning directed annotation of 57 unique lectin specificities. *ACS Chem. Biol.* **17**, 2993–3012 (2022).
2. Kobayashi, Y., Tateno, H., Ogawa, H., Yamamoto, K. & Hirabayashi, J. Comprehensive list of lectins: origins, natures, and carbohydrate specificities. *Methods Mol. Biol.* **1200**, 555–577 (2014).
3. Lee, K. *et al.* Structure of a bimodular botulinum neurotoxin complex provides insights into its oral toxicity. *PLoS Pathog.* **9**, e1003690 (2013).
4. Lee, K. *et al.* High-resolution crystal structure of HA33 of botulinum neurotoxin type B progenitor toxin complex. *Biochem. Biophys. Res. Commun.* **446**, 568–573 (2014).
5. Itoh, Y., Takahashi, K., Satoh, K., Nagao, K. & Fujime, M. Multiplex PCR with confronting two-pair primers for Se genotyping. *International Congress Series* **1261**, 602–604 (2004).
