# Supplementary material for: Gut mucin fucosylation dictates the entry of botulinum toxin complexes
Source: Nat Commun. 2025 Nov 25;16:10442. doi: 10.1038/s41467-025-65384-w (PMC12647801; doi:10.1038/s41467-025-65384-w)
Supplement: Supplementary file 2 — Description of Additional Supplementary Files [file 41467_2025_65384_MOESM2_ESM.pdf]

Description of Additional Supplementary Files

File Name: Supplementary Data 1
Description: List of glycans in the glycan microarray.
