# Supplementary material for: Gut mucin fucosylation dictates the entry of botulinum toxin complexes
Source: Nat Commun. 2025 Nov 25;16:10442. doi: 10.1038/s41467-025-65384-w (PMC12647801; doi:10.1038/s41467-025-65384-w)
Supplement: Supplementary file 4 — Reporting Summary [file 41467_2025_65384_MOESM4_ESM.pdf]

nature portfolio

Corresponding author(s): Yukako Fujinaga

Last updated by author(s): Sep 10, 2025

Reporting Summary

Nature Portfolio wishes to improve the reproducibility of the work that we publish. This form provides structure for consistency and transparency in reporting. For further information on Nature Portfolio policies, see our Editorial Policies and the Editorial Policy Checklist.

Statistics

For all statistical analyses, confirm that the following items are present in the figure legend, table legend, main text, or Methods section.

n/a	Confirmed	
	x	The exact sample size (*n*) for each experimental group/condition, given as a discrete number and unit of measurement
	x	A statement on whether measurements were taken from distinct samples or whether the same sample was measured repeatedly
	x	The statistical test(s) used AND whether they are one- or two-sided<br>*Only common tests should be described solely by name; describe more complex techniques in the Methods section.*
x		A description of all covariates tested
x		A description of any assumptions or corrections, such as tests of normality and adjustment for multiple comparisons
	x	A full description of the statistical parameters including central tendency (e.g. means) or other basic estimates (e.g. regression coefficient) AND variation (e.g. standard deviation) or associated estimates of uncertainty (e.g. confidence intervals)
	x	For null hypothesis testing, the test statistic (e.g. *F*, *t*, *r*) with confidence intervals, effect sizes, degrees of freedom and *P* value noted<br>*Give P values as exact values whenever suitable.*
x		For Bayesian analysis, information on the choice of priors and Markov chain Monte Carlo settings
x		For hierarchical and complex designs, identification of the appropriate level for tests and full reporting of outcomes
x		Estimates of effect sizes (e.g. Cohen's *d*, Pearson's *r*), indicating how they were calculated

Our web collection on statistics for biologists contains articles on many of the points above.

Software and code

Policy information about availability of computer code

Data collection	No software was used for data collection
Data analysis	MetaMorph (Molecular Devices), HKL2000, Molrep, Coot, REFMAC5, PyMOL version 2.4.0 (Schrödinger), QuantStudio Design and Analysis Software version 1.4.3 (Thermo Fisher Scientific), Array Pro Analyzer version 4.5 (Media Cybernetics), R version 4.1.2, FIJI ImageJ version 1.53c.

For manuscripts utilizing custom algorithms or software that are central to the research but not yet described in published literature, software must be made available to editors and reviewers. We strongly encourage code deposition in a community repository (e.g. GitHub). See the Nature Portfolio guidelines for submitting code & software for further information.

Data

Policy information about availability of data

All manuscripts must include a data availability statement. This statement should provide the following information, where applicable:

- Accession codes, unique identifiers, or web links for publicly available datasets
- A description of any restrictions on data availability
- For clinical datasets or third party data, please ensure that the statement adheres to our policy

Coordinates and structure factors of HA3/B in complexes with 3SL and 6SL have been deposited in PDB under accession codes 9UG5 and 9UG6, respectively. All experimental data are contained within the article.

Research involving human participants, their data, or biological material

Policy information about studies with human participants or human data. See also policy information about sex, gender (identity/presentation), and sexual orientation and race, ethnicity and racism.

Reporting on sex and gender	Human samples of intestinal mucins and tissues were collected from 7 male and 3 female.
Reporting on race, ethnicity, or other socially relevant groupings	All samples were provided from Japanese.
Population characteristics	The samples were provided from deceased individuals (age 0-93).
Recruitment	Ten samples were collected within 72 hours post-mortem during routine autopsies at Kanazawa University. One sample (HIM05, 021-650) was excluded from the mucin ELISA because the mucin preparation failed.
Ethics oversight	Human samples of intestinal mucins and tissues were collected from autopsies at the Department of Forensic Medicine and Pathology, Graduate School of Medical Sciences, Kanazawa University. This study was approved by the Human Ethics Committee of Kanazawa University (2022-145).

Note that full information on the approval of the study protocol must also be provided in the manuscript.

Field-specific reporting

Please select the one below that is the best fit for your research. If you are not sure, read the appropriate sections before making your selection.

☒ Life sciences ☐ Behavioural & social sciences ☐ Ecological, evolutionary & environmental sciences

For a reference copy of the document with all sections, see nature.com/documents/nr-reporting-summary-flat.pdf

Life sciences study design

All studies must disclose on these points even when the disclosure is negative.

Sample size	No statistical methods were used to predetermine sample size. Each mice experiment group is consist of 5 or more mice.
Data exclusions	No data was excluded.
Replication	In vitro experiments were conducted at least twice independently. Data are representative of two or three independent experiments.
Randomization	Mice were grouped randomly to conduct the experiment.
Blinding	Administration of toxin were performed by researcher who was blinded to expected outcomes.

Reporting for specific materials, systems and methods

We require information from authors about some types of materials, experimental systems and methods used in many studies. Here, indicate whether each material, system or method listed is relevant to your study. If you are not sure if a list item applies to your research, read the appropriate section before selecting a response.

Materials & experimental systems

n/a	Involved in the study	
☐	☒	Antibodies
☒	☐	Eukaryotic cell lines
☒	☐	Palaeontology and archaeology
☐	☒	Animals and other organisms
☒	☐	Clinical data
☒	☐	Dual use research of concern
☒	☐	Plants

Methods

n/a	Involved in the study	
☒	☐	ChIP-seq
☒	☐	Flow cytometry
☒	☐	MRI-based neuroimaging

Antibodies

Antibodies used	anti-type A botulinum neurotoxin rabbit polyclonal Ab and anti-type B botulinum neurotoxn rabbit polyclonal Ab were generated in Fujinaga's lab 1:2000<br>anti-GP2 mAb (MBL, 2F11-C3) 1:400<br>anti-MUC2 pAb (Abcam, ab76774) 1:500

	Secondary antibodies conjugated with Alexa Fluor 405 (Abcam) or AF 488 (Thermo Fisher Scientific) 1:400<br>anti-FLAG (Merck, M2) 1:10000<br>Secondary antibodies conjugated with horseradish peroxidase (Jackson ImmunoResearch) 1:10000
Validation	Anti-botulinum neurotoxin Abs were validated an ELISA and Western blot in Fujinaga's lab. All other antibodies are commercially available.

Animals and other research organisms

Policy information about studies involving animals; ARRIVE guidelines recommended for reporting animal research, and Sex and Gender in Research

Laboratory animals	7-10 week-old BALB/c (WT) and C57BL6/J (WT and Fut2-null) were used for experiments.
Wild animals	No wild animals were used.
Reporting on sex	The sensitivity to toxin is independent of mouse sex.
Field-collected samples	No field-collected samples were used.
Ethics oversight	All animal experiments were approved by the animal experiment committee of Kanazawa University (AP-163710, AP-214252, AP-163708, AP-214251) and Research Institute for Microbial Diseases (RIMD) of Osaka University (H27-03-0).

Note that full information on the approval of the study protocol must also be provided in the manuscript.

Plants

Seed stocks	N/A
Novel plant genotypes	N/A
Authentication	N/A
